# Unlocking the power of membrane biophysics: enhancing the study of antimicrobial peptides activity and selectivity

**DOI:** 10.1007/s12551-025-01312-y

**Published:** 2025-04-12

**Authors:** Brandt Bertrand, Carlos Munoz-Garay

**Affiliations:** Instituto de Ciencias Físicas (ICF), Universidad Nacional Autónoma de México (UNAM), Avenida Universidad 2001, Chamilpa, 62210 Cuernavaca, Morelos México

**Keywords:** Antimicrobial peptides, Biophysical characterization, Refined lipid models, Membrane properties

## Abstract

The application of membrane-active antimicrobial peptides (AMPs) is considered to be a viable alternative to conventional antibiotics for treating infections caused by multidrug-resistant pathogenic microorganisms. In vitro and in silico biophysical approaches are indispensable for understanding the underlying molecular mechanisms of membrane-active AMPs. Lipid bilayer models are widely used to mimic and study the implication of various factors affecting these bio-active molecules, and their relationship with the physical parameters of the different membranes themselves. The quality and resemblance of these models to their target is crucial for elucidating how these AMPs work. Unfortunately, over the last few decades, no notable efforts have been made to improve or refine membrane mimetics, as it pertains to the elucidation of AMPs molecular mechanisms. In this review, we discuss the importance of improving the quality and resemblance of target membrane models, in terms of lipid composition and distribution, which ultimately directly influence physical parameters such as charge, fluidity, and thickness. In conjunction, membrane and peptide properties determine the global effect of selectivity, activity, and potency. It is therefore essential to define these interactions, and to do so, more refined lipid models are necessary. In this review, we focus on the significant advancements in promoting biomimetic membranes that closely resemble native ones, for which thorough biophysical characterization is key. This includes utilizing more complex lipid compositions that mimic various cell types. Additionally, we discuss important considerations to be taken into account when working with more complex systems.

## Introduction

The biological membrane is of valuable interest to the pharmaceutical industry, as the pharmacokinetics and pharmacodynamics of many drugs are affected or influenced by this semi-permeable barrier. Native membranes are immensely complicated in terms of molecular composition, structural and dynamic properties, and behavior. Biomimetic strategies continue to gain momentum since they resemble fundamental properties and behavior of native lipid bilayers. Moreover, these models are much simpler and easily prepared for in vitro studies (Luchini and Vitiello 2020; Andrade et al. 2021). Lipid membrane biomimetics encompass lipid monolayers, supported bilayers (SBLs), black lipid membranes, solid supported membranes, monolayer cushioned membranes, droplet on hydrogel bilayer, droplet interface bilayers, and liposomes (small, large, and giant); each with distinct physical arrangements, enable the characterization of different biophysical properties of the membrane (El-Beyrouthy and Freeman 2021). These experimental models serve as important tools for understanding the fundamental properties of biological membranes and their associated functions. Each model has its own advantages and limitations, allowing researchers to tailor their approaches depending on the specific aspects of membrane biology they wish to study. The choice of model often depends on the complexity required, the specific scientific question being addressed, and the methods available for characterization and analysis. Properties such as charge, fluidity, and thickness can be tuned at will by modifying lipid composition, which is useful for studying and understanding the interaction between the lipid bilayer and membrane-active molecules such asAMPs (Torcato et al. 2012). Multidisciplinary biophysical approaches that can be employed to gain insight into the molecular properties of membranes and interacting molecules give different information that can be puzzled together to gain a bigger picture (Bertrand et al. 2021). Additionally, the importance given to the models depends heavily on the interest of the investigator. Certain studies may require strict control of physical and chemical parameters, for example understanding the activity and selectivity of AMPs using elaborate/refined membrane biomimetics. On the other hand, peptide folding events or structural transformations can be carried out with micelles or even in the absence of lipids, using solely mixtures of organic solvents and aqueous solution (Ladokhin et al 2010; Alpízar-Pedraza et al. 2024).

In this review we focus on the importance of promoting biomimetic membranes that closely resemble native ones, for which high quality biophysical characterization is necessary. This includes the use of more complex lipid compositions that mimic different cell types. On the other hand, factors and conditions that should be considered when working with more complex systems are discussed.

## Antimicrobial peptides

Even before the discovery of the drug that changed the course of medicine, penicillin, Alexander Flemming noticed bacterial and bacteriostatic activities from nasal secretions of a patient suffering from a common acute cold. The activity of the effector molecule was denominated as lysozyme (antimicrobial enzyme) due to its cell lysing capacity (Nakatsuji and Gallo 2012). It was not until 1939 that the “first true” formal AMP, tyrothricin, was isolated and later tested. In the 1970 s and 1980 s, investigations into insect and anuran AMPs surged (Nassar et al. 2022). Since then, around 4000 proteins with such activities have been isolated from different cells and tissues over a broad spectrum of organisms (Bucataru and Ciobanasu 2024), most of which have been deposited in different databases (Bin Hafeez et al. 2021). AMP database entries continue to grow. Additionally, in most cases, the antimicrobial activity of peptides is used to classify them as AMPs. However, information regarding potential cytotoxicity or hemolytic activity is not always provided. Lysozyme activity was described over 100 years ago (1922), on the other hand, one of the most recent AMP families to be discovered and described is the Achromonodins (Carson et al. 2023). It is now general knowledge that AMPs activities are not limited to inhibiting or killing microbes. Natural AMPs have functional roles including antiviral, antiparasitic, anticancer, insecticidal, spermicidal, chemotactic, anti-inflammatory, wound healing, anti-diabetic, anti-toxin, antioxidant, anti-protease, ion channel inhibitor, among others (Wang et al. 2016).

AMPs typically consist of 12–50 amino acids that play a crucial role in the innate immune system against pathogenic infections in multicellular organisms (Ma et al. 2024). They also provide opportunities for microorganisms competing for nutrients and physical space in microbiological niches. AMPs are generally classified based on origin, structure, biological activity, physicochemical properties, amino acid content/sequence, and mechanism of action (Bin Hafeez et al. 2021). They can share some killing mechanisms of conventional antibiotics, specifically cellular processes involved with DNA replication, transcription, translation, metabolic processes, cell wall synthesis, and an important group has effect on cell membrane perturbation (Talapko et al. 2022).

**Fig. 1:**
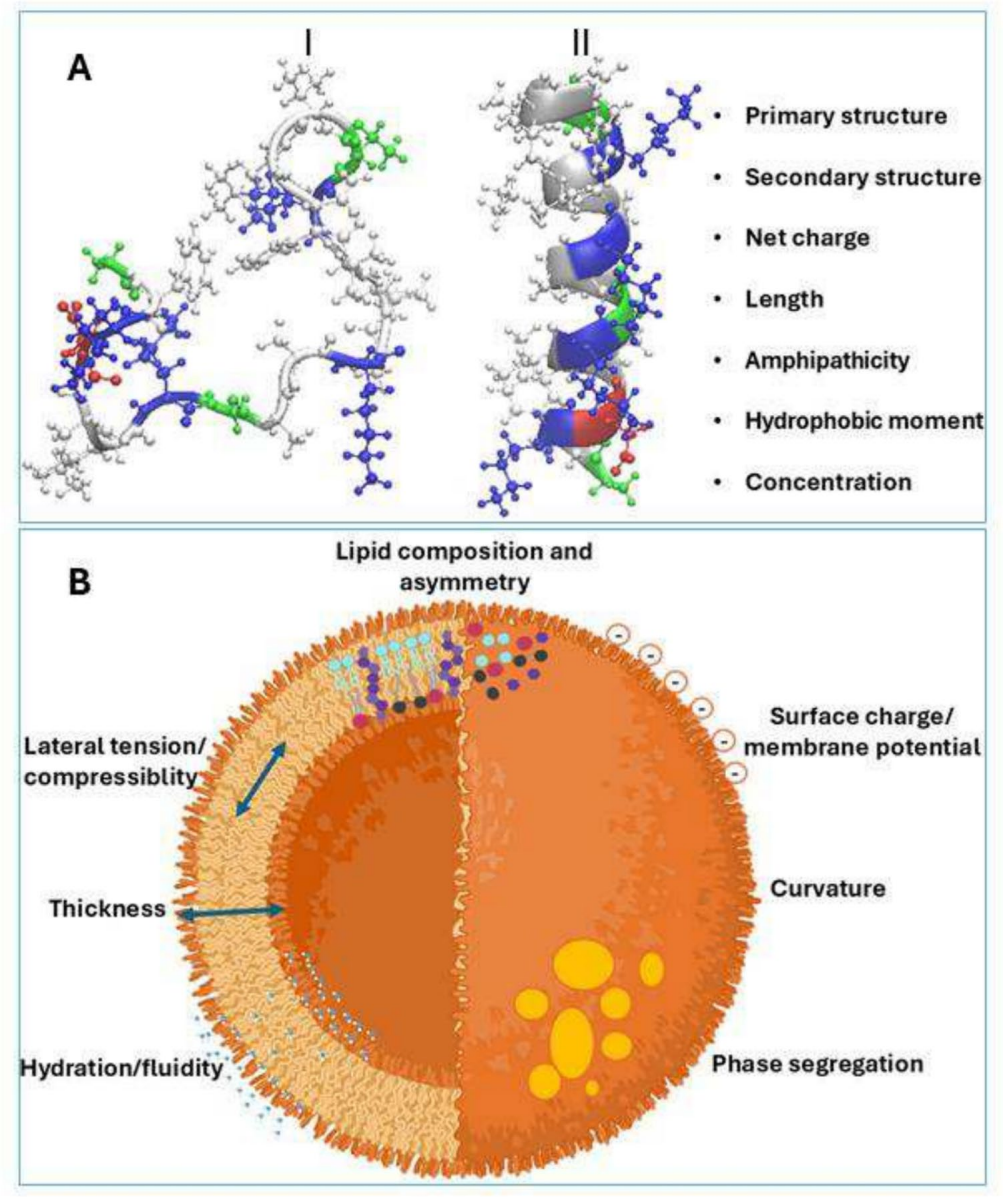
A depiction of the factors that modulate peptide–membrane interaction. **A** Physiochemical properties of AMPs that influence perturbating mechanisms. For example, (I) ascaphin- 8 tends to be denatured in aqueous solution. On the other hand, (II) ascaphin- 8 tends to adopt an alpha-helical conformation in a transmembrane orientation/state. **B** Physicochemical properties of lipid bilayers that influence or determine membrane–peptide interactions. The global result of the relationship between these properties gives rise to peptide activity, selectivity, and potency (image generated with the aid of BioRender)

Cationic alpha helical membrane-active AMPs are the most documented peptides due to their rapid inhibition or killing capabilities, reducing the probability of resistance development by target pathogens. Consensus establishes that basic amino acids such as lysine and arginine (positive charges, near neutral pH) interact electrostatically with the prominently negatively charged head groups of phospholipids of target cell membranes. This initial interaction leads to peptide folding and insertion events, or both can occur simultaneously (Bucataru and Ciobanasu 2024; Agrillo et al. 2023). Depending on peptide properties such as net charge, hydrophobicity, hydrophobic moment, secondary structure (Fig. 1A), and on the other hand, membrane properties such as lipid composition, surface charge, fluidity, curvature, and thickness (Fig. 1B), different perturbation mechanisms may occur. Peptide-lipid interaction may induce different pore structures in lipid bilayers or simply induce micellization (Fong‑Coronado et al. 2024).

The majority of scientific reports on how AMPs work focus mainly on their physicochemical properties and structure, while the properties of the target lipid bilayers have been contemplated to a much lesser extent. It is important to highlight the significance of lipid bilayers characteristics in determining or predicting AMP behavior, particularly with regard to activity, selectivity, and potency, since AMP binding induces substantial changes in membrane structure and organization (Lee et al. 2016). Peptide activity refers to the capacity of the molecule investigated to significantly perturb lipid bilayers, either by pore formation, micellization, or changes in lipid order. Selectivity refers to lipid composition-dependent activity, i.e., loss or gain in activity depending on lipid composition. Potency refers to the affinity or effectiveness of peptide activity, i.e., concentration-activity ratio, whereby peptides that present lower concentrations are considered more potent (Bertrand et al. 2025).

Moving away from the traditional strategies for discovering and characterizing novel antimicrobial peptides, artificial intelligence (AI) methods have experienced a significant surge in the last few years. AI approaches have revolutionized the field of AMP studies by accelerating the discovery of new molecules, since they are relatively quicker and cheaper, compared to traditional means. The main goal of AI approaches for AMP discovery is not only to solve the problem of structure–function relationship, but to be able to predict activity, selectivity, and potency toward different cell types (Brizuela et al. 2025). Currently, amino acid sequences can be predicted accurately as AMPs, or not. However, predicting the activity and potency against a certain pathogen or other cell type has not been solved. Despite significant advances, limitations including challenges in dataset training and missing elements, issues with molecular descriptors based on physicochemical properties, and issues with the learning models themselves, complicate their application in the design of new antimicrobial therapies (Wan et al. 2024). Improvements in experimental aspects and data collection could aid in overcoming limitations in AI peptide science. Existing and future data should be benchmarked, though preprocessing and proper compilation, in order to obtain clean and reproducible data for training datasets. Information on culture conditions such as salt concentration, pH, temperature, culture media, and properties such as stability, solubility, degradation, should also be included or at least considered (Szymczak and Szczurek 2023).

Advances in the understanding of how these molecules exert their biological function could be achieved with innovative approaches that focus on membrane properties and their role, implication, and influence in the overall mechanism of action. Thus, there is a need for adequate lipid bilayer models for studies that facilitate systematic, quantitative analyses, yielding useful results for comparison in literature. This type of information is also relevant for enhancing databases used in many AI applications, which will perform better when they are populated with systematically generated and comparable data.

## Lipidomics

Lipids, along with nucleotides, amino acids, and monosaccharides, are essential building blocks of life on Earth. While there are various examples of cells that lack certain organelles, a nucleus, DNA, or synthesis machinery, there is no known cell type that lacks a lipid membrane (Marth 2008; Deamer 2017). In this sense, lipids have consequential biological roles, stemming from structural, functional, and signaling functions. In order to obtain models that replicate the biological membranes of a particular cell type, it is essential to identify the main lipid components of the cell of interest, especially those that constitute more than 10% of the total lipid composition. This threshold is considered significant, as it may greatly influence the membrane’s macrostructure. Moreover, less abundant lipids also play a crucial role; they can contribute to the cell’s identity in areas such as cellular signaling, regional labeling within the membrane for protein segregation, and other nuanced aspects of metabolic regulation. Therefore, analyses based on lipidomics are invaluable for determining the relevant lipid composition needed to accurately simulate models. Lipid composition determination of native membranes is important to understand their properties in terms of structure, stability, and function of proteins of interest (Moreno et al. 2021). Lipidomics, the large-scale study of pathways and networks of cellular lipids in biological systems is one of the most recent and latest of the omics sciences to gain traction over the last few years, being a branch of metabolomics (Ferreri and Chatgilialoglu 2009). This area of science does not only cover the enzymes in lipid metabolism and transport, but also encompasses lipid-lipid and lipid-protein interactions (van Meer 2005). Additionally, advances in lipid separation methods and mass spectrometry (MS) technologies have allowed for the characterization of the lipidomes of many organisms, and lipidomic profiles differ drastically across different membranes (Maréchal et al. 2011; Symons et al. 2021). An example of contemporary capabilities for determining the lipid composition of tissue is the use of desorption electrospray ionization (DESI), particularly when coupled with tandem MS (MS/MS) for in situ analysis. This approach has the potential to achieve spatial resolution at the level of a single cell (Petras et al. 2017; Bagley et al. 2020; Bodzon-Kulakowska et al. 2020; Iqfath et al. 2024). Reliable, relevant work can now be found that reports the lipid composition of different bacterial strains and cell types (Meetani et al. 2007; Zhang et al. 2011; Parrot et al. 2018; Chen et al. 2024). For example, DESI-MS has been used to differentiate different bacterial species, from Gram-positive and Gram-negative strains. Remarkably, DESI mass spectra can be obtained in less than 2 min (Meetani et al. 2007). Results and analysis of the data showed that growth media played an important role in the quality of the experiments conducted, as spectral fingerprint variation was related to the media preparation procedure. Zhang et al. 2011 also applied DESI-MS to characterize four different bacterial species, including 13 strains of *Salmonella* and two of *Escherichia coli* K- 12. On the contrary to the study carried out by Meetani and colleagues (2007), they concluded that differences in media did not affect their ability to differentiate bacterial species.

To our knowledge, there are no studies of DESI-MS as it relates to the mechanism of action membrane-active AMPs in different cell types. However, a plethora of molecules including antibiotics, peptides, lipopeptides, among others have been discovered using this technology (Li and Li 2022). For example, Bottomley and collaborators (2024) reported the detection of tryptic peptides from tissue sections of mouse and rat brains. Wang et al. 2015 used matrix-assisted laser desorption ionization imaging high-resolution mass spectrometry (MALDI-imaging-HRMS) to identify and visualize the spatial distribution of nine hexacyclopeptides produced by the endophytic fungus *Fusarium solani*. These hexacyclopeptides are communication molecules that are responsible for plant–microbe and microbe-microbe interactions. Thus, this technology has great potential for studying the effects of not only membrane-active AMPs, but AMPs that present other mechanisms of action, on the cellular level. In the near future, the application of advanced lipidomic technologies might just reshape how we view or understand AMPs and their interaction with cytoplasmic membranes.

## Membrane biology

Cell membranes cannot be described merely as boundaries, barriers or envelopes that separate the external environment from the cytosol and its organelles. They not only limit cells but also form external structural extensions such as cilia, flagella, and exons, as well as internal structures like mitochondria, endoplasmic reticulum, Golgi apparatus, and the nucleus, each exhibiting properties specific to these organelles. Cell membranes play vital roles in dynamic cell functions including maintaining structural integrity, homeostasis, cell–cell communication, cell division, regulation and sourcing of substrates for signaling pathways, establishment of specific protein binding sites, cell polarity, and cellular defense among others. These semi/selectively permeable structures are about 4- to 5-nm thick and are composed of lipids arranged in two layers. The external layer or leaflet also known as the exofacial layer is exposed to the surroundings. The inner layer called the cytofacial layer is exposed to the cell cytoplasm. Roughly half the content of cell membranes is made up of lipids (mainly phospholipids). Phospholipids are amphiphilic molecules, containing a hydrophilic head (with variations of phosphate moieties) and hydrophobic acyl chains. This structure is organized in such a way that the hydrophilic heads orient toward aqueous environments, and the hydrophobic tails excluded by water molecules orient toward the tails of both layers forming a hydrophobic core (Dharani 2015; Simons 2016).

Over the decades new developments and discoveries have resulted in our overall understanding of bio membranes, with corresponding adjustments to how they are described, from the early work of Langmuir who coined the term membrane systems, to the semi-fluid dynamics of these structures. The fluid mosaic model introduced by Singer and Nicolson in 1972 described lipids, proteins, and carbohydrates as the primary constituents of the membrane. The most recent and accepted description of bio membranes conserves features of the Singer and Nicolson model, with added concepts such as the lipid-rafts proposed by Simons and Ikonen (Simons and Ikonen 1997). This theory establishes that sterols and other lipids such as sphingolipid clusters present different properties from the rest of the membrane, called microdomains. These “enriched” regions have been shown to be important in numerous cellular processes (Ali and Szabó, 2023). Recent developments in super-resolution imaging such as in situ atomic force microscopy (AFM) imaging and super-resolution fluorescence microscopy (SRFM) have since confirmed the existence of lipid rafts (Zhang et al. 2021).

Given that the primary target of many AMPs is the lipid bilayer, membrane biophysics parameters are central to understanding the mechanism of action of these molecules. According to the cell type, the biophysical properties of this lipid structure are different, as will be discussed later. However, due to historical reasons and the interest in the physiology of ionic flow in nerves (Llinás, 2014) and muscle cell membranes (Alberts et al. 2002), the electrical properties are the best-characterized. In the early stages of bacterial membrane lipid studies *Escherichia coli* was the principal microorganism used as the model. Back then, the results of lipid composition from these investigations were used as the standard for membrane lipid biochemistry. However, with the emergence and development of the genomic era and the consequent growth of lipid inventories it is now general knowledge that there is no such thing as a typical bacterial lipid membrane composition (Parsons and Rock 2013; Sohlemkamp and Gieger, 2016). It is and should be obvious that different bacterial species display different membrane compositions, as they are adapted to different ecological niches (Walczak-Skierska et al. 2024). Moreover, bacterial lipid composition has taxonomic relevance, which is ultimately related to their lifestyle (Sohlemkamp and Gieger, 2016). A single bacterial strain may present variations in the lipid composition of its plasma membrane as a consequence of different growth phases, environmental and nutritional conditions (Sanders and Mittendorf 2011; Ding et al. 2022; Lee et al. 2024). Although there is great diversity of membrane lipids throughout the kingdom of bacteria and ancient bacteria, they generally present abundant phosphatidylglycerol (PG), phosphatidylethanolamine (PE) and diphosphoglycerols (DPG) such as cardiolipin (CL), with phosphatidylcholine (PC), and phosphatidylinositol (PI) and a range of other lipids (example ornithine lipids, glycolipids, sphingolipids, or hopanoids) to a lesser extent (Sohlemkamp and Gieger, 2016).

The human erythrocyte was one of the first mammalian cells (used as a model) to be studied in terms of its lipid composition. In fact, an interesting experiment that demonstrated that the membrane was indeed a lipid bilayer was carried out in red blood cells (RBCs) nearly 100 years ago. Evert Gorter and François Grendel extracted lipids of a defined number of RBCs corresponding to the known surface of plasma membrane. After monolayer experiments, the surface area occupied by the extracted lipids was just about double that of that occupied by the RBCs, inferring that they were bilayers rather than monolayers (Cooper and Sunderland 2000). Mammalian cell membrane lipids are classified into three major groups: glycerolipids, sphingolipids and sterols. Over half of animal plasma membranes are composed of glycerolipids, namely, PC, PE, and phosphatidylserine (PS)(Cockcroft 2021). It should be noted that although PI is represented in a much lower concentration, it is vital for in cell signal processes and the specific segregation of intracellular peripheral proteins (organelle-specific proteins). This phospholipid along with PS are generally distributed on the inner leaflet of these cell membranes, consequently, leading to a greater negative charge on the inside of cells compared to the external/outer monolayer (Cooper and Sunderland 2000). The CLs are a family of negatively charged phospholipids mainly found in the inner membrane of bacteria (El Khoury et al. 2017). They are also present in relatively high concentrations in the mitochondria of eukaryotes, playing a notable role in the function and stabilization of proteins in the bilayer (Yeo et al. 2023). They are associated with lipid microdomain formation located at the cell poles and division plane. CL relocation and clustering have been observed in lipid membrane models mimicking related bacteria (El Khoury et al. 2017). There is also evidence that CL modifies structure and order of lipid bilayers, making them less susceptible to mechanical changes. They have even been shown to hinder transmembrane pore formation (Rocha-Roa et al. 2021). This type of lipid also has the ability to trap protons in an acid anion structure, which gives it the remarkable ability to bind to a large variety of unrelated proteins (Schlame et al. 2008).

## Lipid structure and properties

The most abundant phospholipids in bacterial membranes (PE, PG, and, DPG such as CL present different acyl chains bound to the aforementioned head groups. These hydrophobic regions vary in length (12–20) carbon atoms, with one saturated and one unsaturated fatty acid (Mykytczuk et al. 2007). The acyl chain may also contain cyclopropane fatty acids which are more frequent in Gram-negative bacteria. PE and PG headgroups are usually bound to two acyl chains, while CLs (with two phosphate head groups) are typically bound to four acyl chains (two per headgroup) (Luchini et al. 2021). CLs have a unique dimeric structure, which can have two charges (i.e., one per phosphate group) and, under physiological conditions, can be unprotonated or singly protonated. Relevant in silico advances in lipid force fields, specifically CL, were made about a decade ago, showing a protonation dependent lipid packing (Lemmin et al. 2013).

Sterols, also referred to as steroid alcohols, are rigid compounds with a trans tetracyclic hydrocarbon ring structure. Like phospholipids, they are amphiphilic, possessing a relatively small hydrophilic head containing a 3β-hydroxyl group, and a flexible side fatty acyl chain acting as a tail (Ali and Szabó, 2023). The distribution of sterols is linked to the kingdoms of life (absent in archaebacteria). For example, cholesterol (CHL) can be found in mammalian cells, while ergosterol is confined to fungal cells. Lanosterol can be found in crustaceans, and stigmasterol and β-sitosterol in plants (Cournia et al. 2007; Ali and Szabó, 2023). CHL concentration ranges from 20–50% depending on cell type (Luchini and Vitiello 2020). Its basic function is related to the synthesis of hormones and membrane organization (Cockcroft 2021). Although bacterial species have no sterols, certain species have been found to possess analogs of sterols, likely due to the geometric similarity between these molecules. The hopanoids, present in species like *Methylococcus capsulatus* and *Alicyclobacillus* (formerly *Bacillus*) *acidocaldarius* are believed to conserve the role of sterols in eukaryotes, contributing to stress resistance by tuning membrane rigidity and permeability. Additionally, although they are similar to sterols, they are not functionally interchangeable, as they are not identical. Hopanoids are also related to lipid raft formations. These molecules have other biological roles including nitrogen fixation and plant bacteria association (Belin et al. 2018).

Sphingolipids are known to generally associate with sterols forming microdomains (Bieberich 2018; D’Aprile et al. 2021), but not all species. In a study conducted with the aim of exploring how the nature of the acyl chains of sphingomyelin (SM) influences its lateral distribution in SBLs, confocal microscopy and AFM showed that unlike 16:0 and 24:0 SM, 24:1 SM did not induce phase segregation in ternary lipid mixtures with 1,2-dioleoyl-sn-glycero- 3-phosphocholine (DOPC) and CHL (Maté et al. 2014). This class of lipids is present in relatively lower proportions of cellular lipids, accounting for about 10% of total plasma phospholipids (Calzada et al. 2022). Although they play a role in modulating the physical properties of membranes, they are known to exhibit significant signaling activities. These lipids present a long chain sphingoid base as a backbone, with an amide-linked acyl chain attached instead of an oxygen ester, compared to the glycerol-based phospholipids. Sphingolipid diversity stems from variation in backbone structure, hydrophobic chain length, and the level of unsaturation (Ali and Szabó, 2023).

Lipid geometry is an essential property of these molecules. In general, lipid bilayers are not flat. Lipids that deviate from cylinder shapes contribute to spontaneous curvature. In the case of the phospholipids, PG has a geometric organization resembling a cylindrical shape, as the dimensions occupied by the hydrophobic head group and the hydrophobic region are similar. PE illustrates a narrow-inverted cone, while CL and more broad based inverted conical geometry, since the headgroups occupied a significantly smaller extension in space, compared to the hydrophobic acyl chains (Luchini et al. 2021). Conical and inverted conical lipids induce negative and positive curvature, respectively. Changes in thickness ultimately give rise to a transverse force being exerted in the bilayer.

## Membrane biophysics

Biophysical methods for studying the structure and function of cell membranes as well as related proteins have been extensively reviewed (Torcato et al. 2012; Kwan et al. 2019; Gao and Wang, 2018; Zhang et al. 2021; Andrade et al. 2021, Boulos et al. 2023; Munusamy et al. 2020). These include but are not limited to experimental approaches such as X-ray diffraction, neutron scattering, infrared spectroscopy, nuclear magnetic resonance (NMR), Raman spectroscopy, calorimetry, electron paramagnetic resonance spectroscopy, conventional and advanced fluorescence imaging techniques, electron microscopy, optical and force-based microscopy, and in silico computational approaches mainly molecular dynamics (MD) simulations, and AI.

The nature of lipids that form bilayers, along with their organization, directly influences their physical, mechanical and chemical reactivity, structural and biochemical properties. The best-identified biophysical parameters of lipid membranes are, fluidity, packing density, thickness, selective permeability, elasticity, mechanical stability, bending rigidity, lateral tension, compressibility, curvature, electrical properties (surface charge, transmembrane potential, ionic permeability), lipid phase transitions, lipid flip-flop, lipid raft formation (phase segregation), and asymmetric lipid distribution (Fig. 1B). These parameters are highly interconnected, making the study of their relationships a challenging endeavor for those who enjoy investigation. Achieving a comprehensive understanding of a specific type of membrane requires careful examination of these interconnections. The structural properties consist of thickness, intrinsic curvature, volume/area, and lamellar/non lamellar volume. The mechanical properties comprise bending stiffness, modulus of elasticity, compression, and adhesion. Dynamic parameters for individual lipids include rotation, titling, flip-flop events, and lateral diffusion. The length and degree of saturation of the acyl chains, as well as the alignment of the fatty acid (product of the interaction force with its species and other lipid species) determines thickness and fluidity. Thickness ultimately leads to hydrophobic mismatch between species, causing tilting, stretching and compression. On the other hand, variations in the hydrophilic head groups mediate surface charge and electrostatic interactions primarily with proteins of interest and divalent cations (Janmey and Kinnunen 2006; Lee et al. 2024) (Fig. 1B).

Line tension is also another property that is dependent on lipid composition. This arises when phase segregation events occur influencing lipid packing inside and outside affiliated lipid domains. The line tension along the perimeter of domains represents the energy cost per unit length to build up the interface. This energy is crucial for shaping the heterogeneous surface and defining domain evolution (changes in shape and size) (Rosetti et al. 2017). Related to line tension is lateral pressure that refers to the compression forces at the hydrophilic interface. Membrane elasticity is defined as the resistance to geometric deformation such as bending and stretching forces. The lateral pressure related to fluidity/viscosity, can be considered as the global result of osmotic stress, curvature, hydrophobic mismatches, acyl unsaturation, and phospholipid head interactions (Janmey and Kinnunen 2006). Lateral pressure has been proven to influence peptide insertion into lipid bilayers (Barrera et al. 2012; Morales-Martínez et al. 2022). Peptides are also known to modulate fluidity (Zhang et al. 2024). Specifically, the authors demonstrated (through laurdan generalized polarization (GP), Förster resonance energy transfer, steady-state fluorescence quenching, and MD simulations) that peptide properties (such as amino acid composition, length, and hydrophobicity) can either increase or decrease fluidity of specific bilayers, under certain conditions.

Lipid asymmetry is a relevant factor to consider when studying biological membranes. This terminology refers to the difference in lipid species composition between the outer and inner leaflets. Lipid asymmetry plays a significant role in cellular signaling processes. This asymmetry can also affect lipid organization of each leaflet as it relates to lipid physical states, ultimately influencing phase segregation. Additionally, differences in lipid composition of both leaflets have also been reported to affect lipid-protein interaction (St. Clair et al. 2017). A number of physical parameters of the target bilayer can determine protein behavior, including but not limited to fluidity, curvature, lateral tension, lipid packing, viscosity, charge, bilayer thickness among others (Levental et al. 2020) (Fig. 1B).

Phospholipid headgroup diversity is another fundamental factor to consider when contemplating adequate models. The surface charge should reflect or mimic this property of the native cell. It should be noted that bacteria synthesize different phospholipid head groups to optimize the surface charge. Generally, there must be a balance of zwitterionic phospholipid headgroups such as PC or PE with acidic headgroups such as PG. The optimum distribution of these lipidic species is essential for promoting the correct topology of many integral membrane proteins (Parsons and Rock 2013). Sphingolipids are structural and signaling components of mammalian and yeast cells, but rare in bacteria (Parsons and Rock 2013), thus, this type of lipid is barely considered for mounting bacterial membrane models. On the other hand, there is a remarkable diversity in the fatty acid structures of bacteria. An outstanding difference between Gram-negative and Gram-positive bacteria is that, generally, Gram-negative characteristically produce even-number straight-chain saturated and unsaturated fatty acids, while most gram-positive bacteria predominantly produce odd-number straight-chain saturated and unsaturated fatty acids (Parsons and Rock 2013).

CLs have been shown to increase bilayer thickness by fractions of nanometers. Increased CL concentration has also been linked to increased deuterium order parameter (S_CD_) implying a reduction in area per acyl chain, i.e., increased acyl chain packing (Rocha-Roa et al. 2021). However, our studies have shown that CL under certain conditions (mole ratio and interaction with other lipids) can increase fluidity, instead of reducing it (unpublished data). The discrepancies are most likely due to the molar ratio of increase and the lipid context in which it is added (being abundant in either saturated or unsaturated lipids).

## Current lipid bilayer membrane models

Native cell membranes are often too complex to study not only due to their lipid composition, but other components such proteins, glycolipids, lipopeptides, and other metabolic related molecules (Chan and Boxer 2007). Different cell components and biological phenomena such as exo- and endocytosis, cytoskeleton, membrane proteins, molecular crowding, cell division, protein expression and gene circuitry have been studied using artificial cell mimics (Salehi-Reyhani et al. 2017). In terms of the cellular envelope outer surface components such as surface layer proteins, antifouling components, membrane proteins and channels have also been modeled with advanced technologies (Shen et al. 2014). Biomimetic lipid membranes are synthetic models that hold significant value for both the pharmaceutical industry and academia. These are less complex than native cell membranes, as they focus on the most abundant lipid species. However, they replicate the fundamental environmental and physicochemical properties of biological ones, allowing for the elucidation of the roles of individual components (Chan and Boxer 2007; Carey et al. 2022). Additionally, noise or interference that hinder experimentation and data analyses from other unrelated biomolecules are avoided. These models are relatively easy to prepare and can be studied using various biophysical techniques. This versatility is beneficial for investigating drug-membrane interactions that do not rely on protein receptors. However, they can also be useful in cases involving these receptors, as they help determine the contribution of individual components in the process (Luchini and Vitiello 2020; Andrade et al. 2021).

Lipid membrane mimetics are classified in two principal groups, membranes in aqueous solution and membrane on surfaces. These two approaches require different preparations and biophysical techniques to study their properties. Techniques such as neutron and X-ray scattering, fluorescence spectroscopy, NMR, dynamic light scattering, and calorimetry, among others, are typically used for lipid membranes or particles in solution (for example liposomes). Surface related membranes such as Langmuir monolayers and SBLs are usually studied with technologies such as Langmuir isotherms, which can be coupled to Brewster angle microscopy, attenuated total reflectance- Fourier transform infrared spectroscopy (ATR-FTIR), and advanced microcopy approaches such as AFM, fluorescence and electron microscopy (Luchini and Vitiello 2020).

Liposomes are the most popular bilayer models due to their advantages in lipid biomimetic systems. Liposomes are artificial nanometric lipid vesicles that were first developed in the 1960 s. These vesicles spontaneously form due to the amphiphilic nature of phospholipids. They are typically classified based on size, small unilamellar vesicles (SUVs, 20–100 nm), large unilamellar vesicles (LUVs, > 100 nm), and giant unilamellar vesicles (GUVs, > 1000 nm), or on the number of lamellae, multi lamellar vesicles (MLVs > 5), oligo lamellar vesicles (OLVs, 2–5), or multi vesicular vesicles (MVVs, 1) (Andra et al. 2022). These vesicles can be prepared by reverse evaporation, ethanol injection, thin film hydration, extrusion, electroformation, free-drying, and double emulsion (Zhang et al. 2021).

Single lipid bilayer (i.e., a single lipid species) experiments have been fundamental in understanding membrane properties and peptide interactions. For example, the first studies to demonstrate that tryptophan tends to be enriched at the membrane surface were carried out with a pure DOPC bilayer. Other significant findings carried out with single lipid 1-palmitoyl- 2-oleoylphosphatidylcholine (POPC) bilayers include the work of Bill Wimley’s measurements of hydrophobic scales, which took into account the effect of neighboring residues (White 2023). The now known Wimley–White index describes the partitioning of peptides between water and large unilamellar lipid vesicles, and *n*-octanol. Liposomes are universally accepted synthetic models for cell membranes since they closely mimic the arrangement of fatty acid tails or hydrophobic contents of lipid bilayers (Ferreri and Chatgilialoglu 2009). Although these single lipid bilayer models have provided valuable insights into the mechanisms of action of AMPs, they are deficient in many aspects. For example, single lipid bilayers made up of lipids such as POPC, dimyristoylphosphatidylcholine (DMPC), 1-palmitoyl- 2-oleoyl-sn-glycero- 3-phosphoglycerol (POPG), 1,2-dioleoyl-sn-glycero- 3-phosphoglycerol (DOPG), among others, do not reflect the physical and chemical properties of native lipid membranes. In the case of PC bilayers (which are zwitterionic and thus present no net surface charge), mammalian membranes may be somewhat represented, since they present neutral to slightly positive surface charge (Chen et al. 2016). However, thickness or fluidity, would not be precisely represented, since PC single lipid bilayers are relatively very fluid membranes compared to other systems of different mixtures such as PC/CHL or PC/CHL/SM (Filippov et al. 2003; Vázquez et al. 2021). PG is known for its stabilizing capacity (Zhao et al. 2008). However, in the case of bilayers that present solely negatively charged lipids such as PG, there are still debates on whether electrostatic repulsion between negatively charged head groups would affect packing density (van Uitert et al. 2010; Nowotarska et al. 2014). These simple models are useful for studies focused on protein structural conformations and function in bilayers (ionic channel reconstitution and phospholipase activity) (Morera et al. 2007; Holme et al. 2018). Unlike detergent micelles, organic solvent, aqueous solution mixtures that mimic the amphiphilic nature of lipid bilayers to explore structural changes, liposomes are highly recommended because they consist strictly of a bilayer (Gopal et al. 2012). Avitabile and collaborators (2014) went further with their circular dichroism (CD) analysis evaluating AMP structure in the presence of native cells. They argued that structural behavior of peptides with model membranes may not accurately represent conformation changes with the bacterial envelope. This is because AMPs may previously interact with the lipopolysaccharides, porins, peptidoglycan, teichoic acid or lipoteichoic acids that cover the Gram-negative and Gram-positive bacterial cells. In fact, there is evidence that suggests that peptides form aggregates on the outer leaflet, before passing through by self-promoted uptake to reach the cytoplasmic membrane (Avitabile et al. 2014).

Binary lipid mixtures have been exhaustively studied by a plethora of biophysical experimental techniques and corroborated by MD simulations. These mixtures include PE/CL, PC/CL, PE/PG, PG/CL among others (Luchini et al. 2021). In terms of mammalian systems, PC/CHL, is the most common mixture. Although CHL and SM have been shown to induce phase segregation in membrane models, in our first-hand experience we have noticed that properties such as fluidity (as determined by laurdan GP) are not severely affected. In the case of specific peptides such as pandinin- 2 that we have investigated, there is no significant difference in activity between PC/CHL and PC/CHL/SM/PE bilayers (Bertrand et al. 2020). The PC/CHL binary system has been used successfully to study the effect of CHL on peptide activity by varying its concentration, as in the case of the AMP DD K isolated from the skin of the amphibian *Phyllomedusa distincta* (Verly et al. 2008). The results from this investigation showed that increasing CHL concentration reduced peptide activity. Simple models (single lipid, binary, or ternary) can also be used comparing peptide activity of different molecules, such as peptide derivatives, with the aim of studying protein parameters such as peptide charge and hydrophobicity through liposome permeabilization and fluidity experiments (Oñate-Garzón et al. 2017). In this sense, historically, simple models have been proven to be useful. However, it should be noted that these results cannot be translated directly to activity and selectivity against native cells. Many times, even with two or three major lipids present in the native cell, parameters such as fluidity are significantly different from those of biological membranes. For this reason, it is essential to provide, as much as possible, the physical parameters of the proposed model in order to gain a deeper understanding of the system, beyond merely the lipid proportions used.

1-palmitoyl- 2-oleoyl-sn-glycero- 3-phosphoethanolamine (POPE) and POPG lipids have been popularly used to model *E. coli* membranes. However, in a study conducted by Lopes and collaborators (2011) that contemplated the importance of CL in natural membranes, they showed that the properties of a ternary mixture of POPE/POPG/CL were similar to the natural polar and total extracts of these bacteria. The validation of their systems was corroborated through dynamic light scattering, and steady state fluorescence anisotropy using fluorescent probes that report on different membrane regions. As a complementary technique, AFM was employed. In another relatively recent study, Luchini and collaborators (2021) pointed out the importance of designing drug-testing platforms such as liposome models, since they are crucial for understanding drug-membrane interactions at the molecular level. Small-angle neutron scattering, dynamic light scattering, and electron paramagnetic spectroscopy were used to characterize their models made of PE, PG, and CL. Their general conclusions were that CL induces thicker bilayers, gave rise to higher curvature, and produced increased fluidity. Vesicle core radius and hydrodynamic size were also different in function of CL concentration.

## Limitations of lipid bilayer membrane models and other considerations

Artificial models such as vesicles for the biophysical characterization/description of membrane structure and function, and the effects of disrupting agents like AMPs have been widely used and accepted (Epand and Epand 2003). Although lipid biomimetic has been fundamental in our current understanding of membrane and membrane-active peptides biophysics, they present various disadvantages. Firstly, these models as with other models do not mimic the whole complexity of native ones. They are also generally void of other non-lipidic membrane components that may or may not be relevant for the molecular processes being investigated. Disrupting agents are typically tested in microbiological or cytotoxic assays over extended periods of time (hours and even days), while liposome experiments are short lived (second and minutes). Another limitation of these versatile systems is that they are “naked” structures and are exposed to higher rates of lipid oxidation (Andrade et al. 2021).

Native biological membranes are asymmetrical. However, most literature on liposomes involves symmetrical vesicles (Gardea-Gutiérrez et al. 2023). Mimicking lipid bilayer asymmetry is not an easy task. Although the majority of liposomal studies used symmetrical models, there have been notable efforts to improve asymmetric biomimetics (Andrade et al. 2021). Generating asymmetric liposomes implies added complexity to formulation protocols. Moreover, this system lacks standardization in terms of synthesis and characterization methods, since formulation methods are not reproducible and asymmetry is difficult to characterize (Gardea-Gutiérrez et al. 2023). These procedures involve the use of carrier proteins such as bovine serum albumin and pro-sterol carrier protein (pro-SCP2). Reverse-phase evaporation and microfluidics jetting have also been implemented to produce these types of liposomes. Asymmetric vesicles can also be synthesized using pH gradients and water-in-oil based techniques. However, the latter complicates liposome quality due to residual oil contamination (Gardea-Gutiérrez et al. 2023). Additionally, enzymes that selectively modify outer leaflet lipid headgroups have also been employed. Some examples of these enzymatically catalyzed assays for asymmetric liposomes include the use of PS-decarboxylase to convert PS of the outer leaflet to PE, and phospholipase D to modify PC of the outer leaflet to either PE or PS. A group of molecules known as cyclodextrins have the capacity to modify membrane lipid structure of cells and models. They are composed of glucose molecules bound by glycosidic bonds into macrocycles-forming structure with a hydrophilic outer surface and a central hydrophobic pocket that accommodate a lipid chain. Members of this class of molecules have different affinities of different lipid species, resulting in varying activities. For example, methyl-β-cyclo dextrin (mβCD) can completely dissolve lipid vesicles at certain concentrations, while hydroxypropyl-α-cyclodextrin (HPαCD) selectively interacts with phospholipids, but not CHL (Huang and London, 2013). There are published reports of exchanges so efficient that it is possible to replace almost entirely the population of outer leaflet phospholipids with exogenous lipids in cells and model membranes (Suresh and London, 2022).

One of the most significant advances in asymmetric vesicles was developed by Doktorova and colleagues (2018) which contributes significantly to the development of the next generation of cell membrane biomimetics. They produced asymmetric vesicles by mβCD catalyzed exchange of the outer lipids between liposome pools of different composition. Not only did they generate these asymmetric vesicles but also quantified the lipid composition of each leaflet via the NMR lanthanide shift reagent Pr^3+^. The protocol yields large amounts of liposomes in approximately 12 h. It can act as a donor or acceptor depending on the dynamic equilibrium that exists between intact vesicles and mβCD/lipid complexes. A major disadvantage of this approach is the purification of liposomes of interest, which must be done by phase separation after ultracentrifugation. Compounds in the phase where the asymmetric liposomes are found may contaminate them, leading to issues such as osmotic stress affecting membrane properties (Doktorova et al. 2018). It must be noted that, in their protocol there are numerous critical steps that must be considered, from the preparation of donor and acceptor films and vesicles, to the incubation of mβCD, resulting vesicles, and sample analysis.

The work of Kakuda and colleagues (2021) exemplifies the importance of the use of asymmetric vesicles in membrane-active protein studies. Particularly, they investigated the effect of asymmetry in lipid vesicles on the pore-forming toxin, perfringolysin O (PFO). They demonstrated that lipid composition and organization not only affect the interaction with their lipid models, but also insertion into the bilayer, structural changes, and oligomerization. The results of their investigation generated further questions. For example, how can the headgroups of the inner leaflet influence PFO insertion? Among other questions that arose, one was the other way around; how does the pore-forming toxin affect lipid asymmetry? Marx and colleagues (2021) also evaluated the effect of membrane asymmetry on the activity of various AMPs, namely L18 W-PGLa, magainin 2 (MG2a), and a derivative of lactoferricin. They concluded that the increased vulnerability of their asymmetric liposomal preparations was due to tension differences between the compositionally distinct monolayers, and not because of increased peptide partitioning.

In another interesting, relevant and relatively recent study Vázquez and collaborators (2021) investigated bilayer asymmetry using AFM imaging and force spectroscopy. They assessed domain formation to study the nanomechanical properties of asymmetric SLBs mimicking membrane rafts. Their models were prepared by incorporating N-palmitoyl-sphingomyelin (16:0SM) into the outer leaflet of previously formed DOPC/CHL SLBs through mβCD-mediated lipid exchange. Significant differences in size and morphology of the domains of symmetric and asymmetric bilayers were observed and characterized. Detailed nanoscale characterization of their asymmetric SLBs contributes to our understanding of membrane biology, as a more realistic model for studies pertaining to membrane properties and protein function, or how lipid asymmetry could modulate the interaction of molecules such as AMPs. To circumvent these limitations and difficulties asymmetrical planar bilayers were developed initially using the Langmuir/Blodgett technique. A representative case of this technique was published by Michel and collaborators (2017) where they mimicked the asymmetric outer membrane of Gram-negative bacteria using phospholipids and lipopolysaccharides. Implementing the Langmuir–Blodgett/Langmuir–Schaefer technique to generate the asymmetric lipid bilayer. The authors demonstrated how the properties of the asymmetric scaffold structure allowed discrimination of two structurally related peptides, one active and the other inactive. Neutron reflectometry and AFM revealed how the active peptide exerted its activity through the carpet mechanism, ultimately removing lipids from the membrane surface. On another note, the use of ghost cells, such as erythrocyte ghosts have also been used since the cell membrane context is native. (St. Clair et al. 2017).

In the last few decades lipidomic studies with the aid of other technologies such as gas chromatography have exponentially expanded our knowledge on lipid composition of cell membranes, and concomitantly aided our understanding of physical properties and processes, and their metabolic implications. Although scientific research continues, there are still organisms where information is limited. For example, *Trypanosoma cruzi* is a medically relevant human parasite that causes Chagas disease, and for which, up to date there is no effective antiparasitic drug-treatment (Tarleton 2016). There is substantial data on total lipid content and lipid metabolism in *T. cruzi* at different stages of its life cycle, and its relationship with the host. However, the distribution of its lipids, in terms of membrane asymmetry, is not clear (Contreras et al. 1997; Booth and Smith 2020). Although glycerolipids such as PI and PS are known to be prominently distributed in the inner leaflet (Vahedi et al. 2020), the exact ratios are unknown. Thus, attempts to model this parasite’s plasma membrane are burdened with a certain level of uncertainty.

Another aspect that should be considered when contemplating refining or improving plasma membrane models is how important it is to include all the known lipid species. For instance, the CHARMM-GUI webserver (Jo et al. 2008), which is a platform that has substantially facilitated mounting membrane models for in silico analyses has an archive with 18 biomembranes including mammalian plasma model, as well as a number of organelles, fungal, algae plasma membranes. Each one in this repository consists of over ten lipidic species, some of which represent less than 1% of the total lipid content. Perhaps performing in silico studies with all the reported lipid species is feasible. On the other hand, it is not practical to prepare lipid models such as liposomes, SBLs or monolayers in which all the lipid species are represented. This is because the mole ratios of certain lipids are so small that their effect on the macrostructure may be insignificant. Furthermore, lipids that are represented in extremely low quantities have significant roles in cell signaling processes (Rodas-Junco et al. 2020; Xing et al. 2021), rather than the physical properties of the membranes in which they are found. In this sense, the application of membrane mimetics to study the effect of AMPs may not be affected by the exclusion of lipids that are not abundant in the native cells.

In the case of bacterial membrane mimetics, a component that is hardly ever contemplated is lipid species pertaining to the hopanoid family. A limitation of using this lipid structure is that it is not a common product found as a reactive. There are only a few companies that commercially produce this compound, and it is quite expensive. Another factor that increases the complexity of the problem is that hopanoids can have different levels of glycosylation that give great diversity (Komaniecka et al. 2014; Abdel-Mawgoud and Stephanopoulos 2018). An option for mimicking lipid bilayers is to purchase cell membrane lipid extracts. However, the available bacterial extracts are generally from model organisms like *E. coli*, thus, hopanoids like diplopterol are not present in these mixtures. To circumvent this issue the use of CHL (sterol present in animal cells) has been used to simulate the surface of Gram-positive bacterial cell membranes (Wang et al. 2021). The strategy addresses an aspect related to the properties of the membrane being simulated but complicates data interpretation by including a lipid not specific to bacteria.

LUVs are generally the most common in AMPs studies, SUVs and GUVs are reported to a lesser degree (Tamba and Yamazaki, 2005; Carvalho et al. 2008; Espeche et al. 2024). LUVs and GUVs are considered the most adequate models, unlike SUVs. Although SUVs have also contributed immensely to knowledge on AMPs through biophysical characterization, it has been debated that their size contributes to a major flaw. Presenting sizes of less than 100 nm, on average 20 nm, bilipid curvature is greater than that of LUVs and GUVs, thus, the lateral pressure is greater. Their high surface curvature can lead to distorted lipid packing and consequently to unstable structures (Torcato et al. 2012). This result of increased tension in addition to smaller vesicle size has been shown to reduce the ability of peptides to form pores activity (Nir and Nieva 2000). SUVs are also relatively unstable vesicles compared to LUVs and GUVs. Smaller vesicle size possesses larger tension, and on contact, they tend to destabilize immediately in order to reduce the tension (Lin et al.^2011^), and not necessarily due to protein-membrane specific interactions.

It should also be noted that, although increased complexity in model bilayer composition allows for certain advantages, there are disadvantages that cannot be ignored. For example, analyses such as ATR-FITR that have the precision of identifying functional groups of all molecules present that are IR active become an overwhelming task. In this sense, simpler models such as single lipid or binary systems are favored, as relevant information from lipid molecules such as the hydrophilic head groups and hydrophobic chain, and their interaction with peptide molecules is less complicated.

**Fig. 2:**
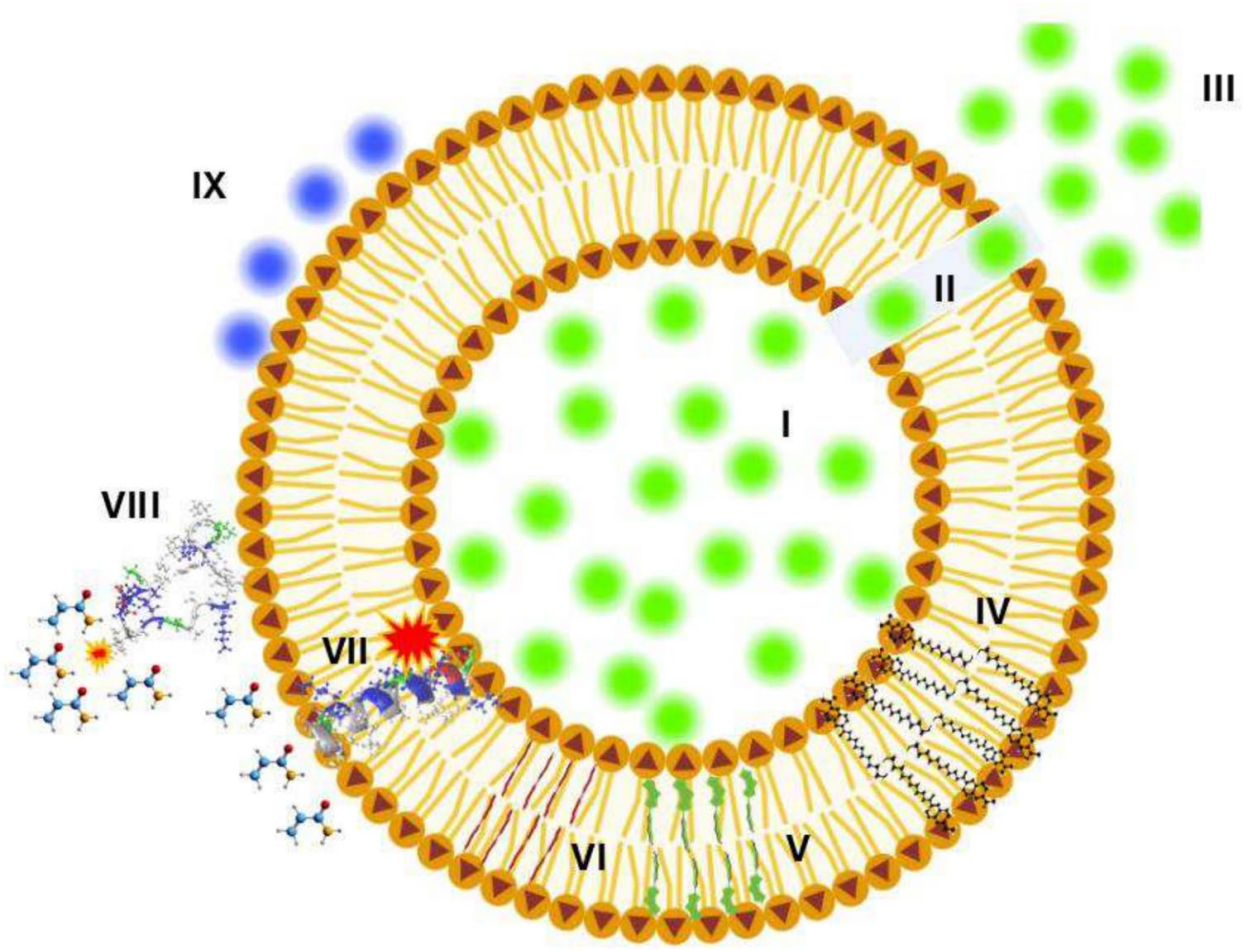
Fluorescence spectroscopy for studying AMP-induced changes in membrane biomimetics. (I–III) probe encapsulation with self-quenching, leakage through defects, and dilution with subsequent dequenching. (IV) Solvatochromic probes, such as embedded in the bilayer gives information on water permeation into the hydrophobic core. (V-VI) Planarizable push–pull fluorescent probes that suffer changes in fluorescence spectrum. Molecular twisting (V, green) with reduced lateral pressure and flattening (VI, red) with greater lateral pressure. (VII–VIII) Intrinsic AMP fluorescence, where greater fluorescence can be observed with peptide in the inserted state and in the absence of hydrophilic quencher/molecules with grey, blue, red, and yellow spheres (VI). Lower fluorescence due to the quenching of intrinsic fluorescence by quencher in the solution. (IX) probes used for studying voltage changes of lipid bilayer models (Image generated with the aid of BioRender)

The use of fluorescent probes for understanding membrane dynamics and protein interactions has been a pillar in this area of biophysics. They are used to monitor numerous cellular processes in real time and in situ. Moreover, application is relatively easy, they present high sensitivity and are generally non-invasive (Tong et al. 2024). Figure 2 depicts the application of a few examples of fluorescent molecules used in membrane science, and to gain insight into how small molecules such as AMPs can modify important properties. Fluorescent probes used to specifically study changes in membrane properties induced by AMPs can be characterized based on environment-sensitive fluorophores, such as polarity sensitive solvatochromic dyes, viscosity-sensitive fluorescent molecular motors, mechanic sensitive fluorescent flippers, and dyes which change their spectral properties in response to voltage variation (Liu et al. 2020; Klymchenko 2022).

One of the most popular strategies for studying AMP activity in membranes is by evaluating lipid bilayer integrity (i.e., permeability). Self-quenching dyes such as calcein and carboxyfluorescein can be encapsulated at high concentrations within lipid vesicles (liposomes). Significant perturbation (pore formation, micellization, etc.) that results in dye leakage and dilution in the external medium, leads to increased signals that can be measured through fluorescence spectroscopy (Grad et al. 2022) (Fig. 2, I–III). This approach has been used to study the effect of different membrane-active AMPs and their synthetic derivatives (Ambroggio et al. 2005; Haney et al. 2012; Arias et al. 2018; Morales-Martínez et al. 2022). Efficiency and selectivity can also be calculated from dose–response curves and leakage kinetics (Sorochkina et al. 2013; Nasr et al. 2020). Apart from peptide activity, the effect of membrane surface charge and fluidity on peptide behavior can be investigated (Verly et al. 2008; Morales-Martínez et al. 2022; Giraldo-Lorza et al. 2024). In our workflow, this is the first main test for determining bilayer perturbing activity of peptides and other molecules. It is important to note that this method does not give information on the mechanism of perturbation (i.e., pore formation, micellization), only the presence or absence of such activity (Ambroggio et al. 2005; Bertrand et al. 2021). Some dyes lack self-quenching properties. In these cases, fluorescent probes are encapsulated with quenchers. An example is the 8-aminonaphthalene- 1,3,6-trisulfonic acid-*p*-xylene-bis-pyridinium bromide (ANTS-DPX) assay. The cationic DPX quenches the polyanionic dye’s fluorescence when encapsulated. On leakage and dilution, the quencher loses its capability of interfering with the fluorophore (dequenching), thus, increased fluorescence is observed (Aguilera et al. 2021). On the other hand, polymers of different molecular weights can be conjugated to fluorophores, for example fluorescein isothiocyanate (FITC) dextran and Texas-Red dextran. The leakage of these conjugates from liposomes or cells can give rough estimates of pores or channels formed through bilayer lipid structures (Uematsu and Matsuzki, 2000; Tamba et al. 2010; Bertrand et al. 2020).

Laurdan (6-dodecanoyl- 2-dimethylaminonaphthalene) has been widely used to study fluidity/rigidity/hydration of native and membrane biomimetics. The fundamentals of how the technique works have been widely revised (Harris et al. 2002; Jay and Hamilton, 2016; Scheinpflug et al. 2017; Gunther et al. 2021). The general concept is that this solvatochromic fluorophore responds to lipid bilayer hydration levels. Increased hydration can be interpreted and greater area per lipid, greater lipid lateral movement or fluidity. In some cases, interaction, and especially peptide insertion may result in shifts in fluorescence spectrum which can be interpreted as changes in lipid compactness, or even lipid order phase changes (Ma et al. 2018; Zorilă et al. 2020) (Fig. 2, IV). It is important to note that not all membrane-active peptides show or induce changes in laurdan GP. Another similar fluorophore used on this basis is Prodan (6-propionyl- 2-dimethylaminonaphthalene). Prodan is located close to the surface while laurdan allocates deeper into lipid bilayers (Jay and Hamilton 2017). The combination of these two fluorophores gives even more detailed information on fluidity (Ghazaryan et al. 2015). Interestingly, these dyes can also be used with native cells such as bacteria, fungi and mammalian cells. Perhaps, an effort to replicate the fluidity of native cells could be appropriate to improve lipid membrane models.

One of the most recent advances in terms of fluorescent probes for membrane biophysics is the development of the mechanical sensing dyes. They are also known as “flipper” molecules due to their capacity to flatten or twist depending on lipid packing. These mechanical changes result in red or blue shifts, respectively. Flipper RT is a planarizable push–pull fluorescent probe that responds to changes in lipid order, membrane tension, osmotic pressure of both native and membrane biomimetics, and the effect of different molecules and environmental factors (Colom et al. 2018) (Fig. 2, V–VI). Given all the previously mentioned sensing capacities of this class of probes, it promises to be useful for controlling and tuning features of these models.

The interaction of AMPs with lipid bilayers can be studied using the intrinsic fluorescence of these proteins. Aromatic amino acids, tryptophan, tyrosine and phenylalanine are naturally occurring fluorophores (Verma et al. 2023). Tryptophan and tyrosine are most used because of their higher quantum yields. AMPs with one or more of these amino acid residues that insert into lipid bilayers can be monitored using hydrophilic quenchers such as acrylamide. Insertion depth or efficiency can be estimated by calculating the Stern–Volmer coefficients. Peptides with reduced capacity to penetrate membranes are exposed, and their fluorescence will be quenched. On the other hand, if the peptide is deeply inserted, the quencher has limited or no access into the hydrophobic core, and therefore, cannot efficiently quench (Jobin et al. 2019) (Fig. 2, VII-VIII). Another approach is to use fluorescently labeled peptides that circumvents disadvantages such as low quantum yields and interference from surrounding amino acid residues (Saar-Dover et al. 2013).

Cyanine of 3,3′-Dipropylthiadicarbocyanine iodide (diSC3 - 5) is also very useful for studying membrane properties and the effect of molecules that interact with these structures. Cyanine is mostly used to estimate membrane potential or study depolarization events in cells (Peña et al. 1984; Buttress et al. 2022), although it can be used in liposome assays (Waggoner 1979; Letellier and Shechter 1979). As this parameter depends on membrane impermeability (i.e., separation of internal and external ionic conditions), the perturbing/depolarizing effect of AMPs can be studied (Wu et al.^1999^). This cationic dye electrostatically deposits onto the negatively charged surfaces of bacterial cells or liposomes, resulting in self-quenching. Loss of membrane potential (depolarization) due to perturbation results in an increase in fluorescence (Sims et al. 1974) (Fig. 2, IX). TMRE (tetramethyl rhodamine ethyl ester, perchlorate, Biotium) or TPP (tetraphenylphosphonium chloride, Aldrich) are also used to estimate membrane potential (Smirnova et al. 2018). These membrane potential/depolarizing assays are used to complement dye leakage assays.

### Molecular dynamics simulations

Over the last 50 years, liposomes have been studied mainly through in vitro biophysical sciences. However, recently, MD simulations of these synthetic vesicles have been gaining interest as a new tool that can provide insight into molecular and cellular biology phenomena. Additionally, knowledge on stability and formation mechanisms can aid in the rational design of these vesicles. For instance, computation methods have aided in understanding the behavior of lipids within asymmetric liposomal formulations, as well as predicting their stability, compatibility with the charge and the solvent, and even their pharmacokinetic behavior (Gardea-Gutiérrez et al. 2023). The main advantage of liposome MD is the screening capacity and decreased costs. These studies span from the spontaneous formation of vesicles starting with phospholipid species in aqueous solutions to properties and behavior of pre-formed liposomes (Marrink and Mark 2003; Risselada and Marrink 2009; Hashemzadeh et al. 2020), to the interaction of liposomes with membrane-active molecules such as AMPs. In the case of self-assembly, phospholipid geometry influences the formation of bilayers consistent with literature (; Parchekani et al. 2022; Gardea-Gutiérrez et al. 2023).

MD simulations may be carried in all-atom (AA) or in coarse-grained (CG) configurations. AA MD simulations are useful for studying the structure and dynamics of biomolecules. However, representing every atom is computationally costly which ultimately limits the size and timescales of these systems (tens-hundreds of nanoseconds). On the other hand, CG simulations were developed to circumvent challenges of AA MDs. Groups of atoms are represented as beads (Perlmutter et al. 2011). This lowers computational calculation costs by speeding up MD experiments. This results in the possibility of longer time scales (tens to hundreds of microseconds) but with lesser details. It is important to note that currently CG configurations are in good agreement with atomically detailed simulations (Balatti et al. 2017). Most in silico studies of liposomes have been carried out using CG configuration due to the number of lipids required to form stable liposomes (generally over 2500 lipids) (Jämbeck et al. 2014). The number of atoms in these systems is generally greater compared to planar bilayer patches used in MDs. Additionally, with an increased number of lipid atoms, the number of water molecules also are greater. The behavior of water molecules depends on their immediate environment (i.e., at the membrane interface or in the bulk solution) (Calero et al. 2016). Thus, the number and position of water molecules of a MD simulation can ultimately have an impact on MD simulations (i.e., ns/day). Up to date very few in silico studies have been carried out studying the effect of AMPs on liposome structure and integrity. Most studies have focused on drug delivery applications (Lee 2020).

Risselada and Marrink (2009) studied the curvature effect on lipid packing and dynamics in symmetric and asymmetric liposomes via CG MD simulations. Overall, they showed that curvature induced thinning and reduced the thermal expansivity of the bilayer. Additionally, their data revealed that in the inner monolayer, the lipid head groups were closely packed and dehydrated, with relatively disordered lipid tails, compared to the outer monolayer. Also, higher temperatures favored more equally populated monolayers. Furthermore, they discussed how differences in lipid composition in the different monolayers affect membrane properties and dynamics. Studies like these contribute to the understanding of the dynamics of these systems, and aid in better application and design of lipid models. In one particularly interesting study carried out by Bond and collaborators, the membrane-active AMP Maculatin 1.1 was investigated with respect to its interaction with liposome models in silico (Bond et al. 2008). Eight microseconds of CG MD simulations with a zwitterionic phospholipid model at different peptide/lipid ratios revealed peptide aggregation and spontaneous cooperative insertion. Additionally, four monomers were the minimum number for transmembrane oligomerization. The main conclusion of that paper indicated that the mechanism of action of this peptide was likely not pore formation due to the absence of a simple and well-defined central channel, and exclusion of phospholipid headgroups from the oligomer. On another note, attempts to mount liposomes in AA configurations in our research group using the CHARMM-GUI online platform have proven to be futile, probably due to system size; however, CG configurations have been mounted without any setbacks.

**Fig. 3:**
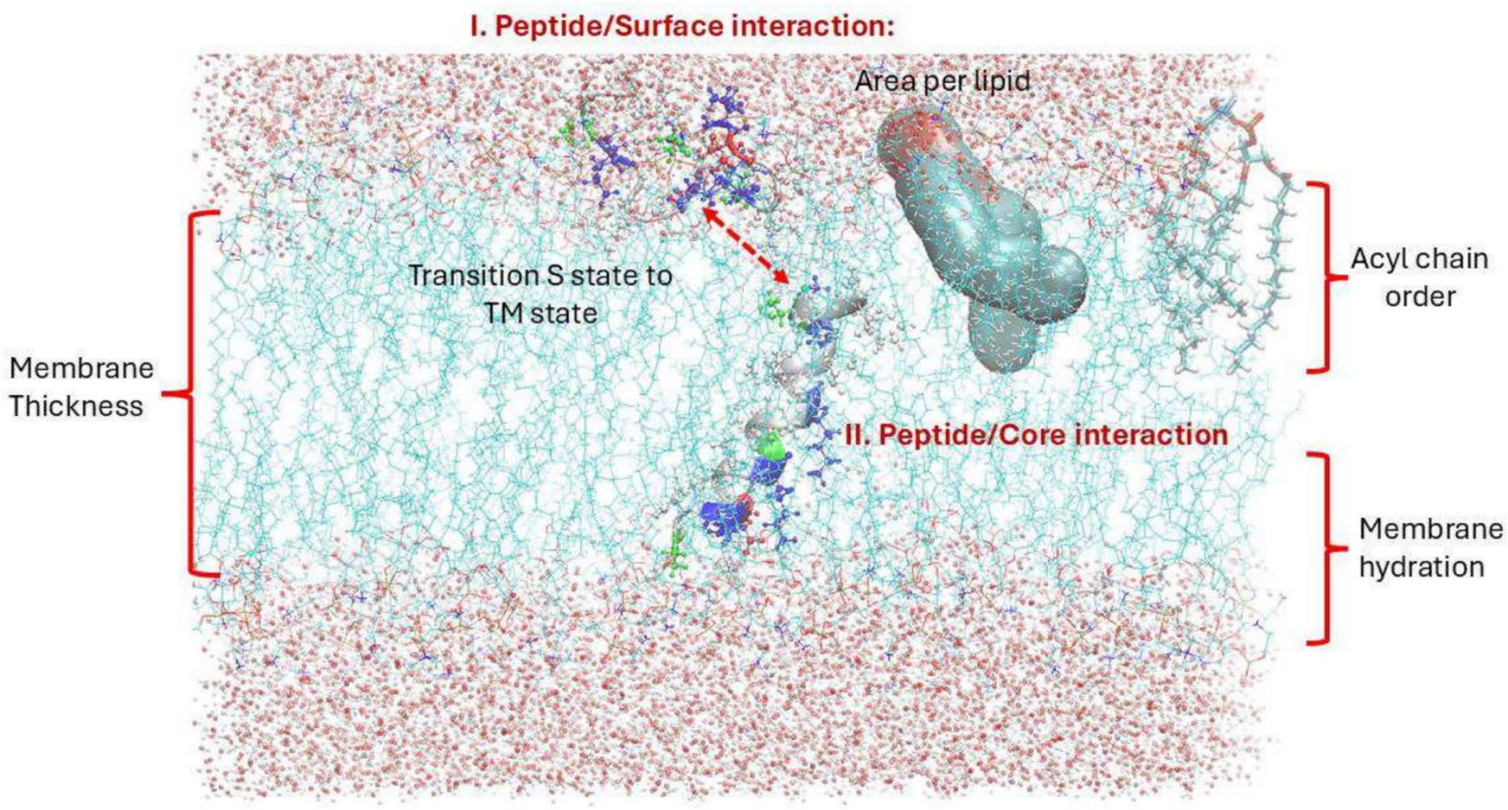
MD simulations of the interaction of AMPs with bilayer lipid bilayer models. (I) Initial interaction of peptides with the membrane surface through analyses including distance from the surface, number of contacts and hydrogen bonds, solvent accessible surface area, and energetic assessments. (II) Peptide interaction embedded in the hydrophobic core, including effect on membrane parameters such as thickness, area per lipid, acyl chain order, and hydration levels. Additionally, peptide folding, oligomerization, and pore-forming events can be evaluated

For in silico analyses of lipid bilayer models and the interaction of AMPs, as in the case of in vitro studies, the effect on parameters such as thickness, area per lipid, acyl chain order, and lipid bilayer hydration are some common analyses reported (Leontiadou et al. 2006; Arasteh and Bagheri, 2016; Jobin et al. 2019; Maleš and Zornic, 2022; Frazee et al. 2021; Kumar et al. 2024). On the other hand, peptide interaction studies encompass surface interactions such as distance from surface, number of contacts, and hydrogen bonds, solvent accessible surface area, and energetic assessments (Fig. 3). It is noteworthy that an important aspect is the transition from the soluble or surface (S) state to the inserted transmembrane (TM) state (La Rocca et al. 1999; Sengupta et al. 2008). Once inserted into the membrane, peptide folding events, oligomerization and pore formation alongside energetic assessments, are of great interest (Wang et al. 2014; Ulmschneider and Ulmschneider 2018; Tyagi et al. 2019; Ermakova and Kurbanov 2023). These complex and intertwined properties between AMPs and membranes for interaction and activity highlight the importance of focusing not only on the proteins of interest, but equally important, the membrane target. A clear example of the importance of membrane properties on peptide interaction can be seen in the work published by Su and colleagues (2020), where they showed that the peptides investigated (BP100, MSI- 103, and MSI- 78) had a clear preference toward the liquid disordered phase compared to the liquid ordered phase. Compared to the in vitro methods for preparing asymmetrical membrane models, the in silico approach is straightforward, since the exact compositions of the outer and inner monolayers can be strictly controlled.

Recently, MD simulations of more complex membrane systems have been gaining terrain in the field of membrane-protein biophysics. Such is the case of the work published by Sharma and Ayappa 2022 that modeled the translocation of the AMP CM15 through the outer membrane of Gram-negative bacteria via atomistic MD and umbrella-sampling simulations. This complex system consists of tails of the LPS (Lipid-A) that mirror the hydrophobic tails of the inner leaflet of the outer membrane. The lipid-A molecules are covalently bonded to the core saccharides, which themselves are bonded to the polymeric O-antigen sugars. The results of their investigation revealed that CM15 translocation across the phospholipid bilayer was complicated due to the secondary structure adopted in different environments. The peptide also bonded favorably with the outermost region of the complex (O-antigen–core saccharides region). However, the authors determined that there was a large barrier at the core saccharides-Lipid-A interface. They pointed out that despite the molecular insight and contribution of their paper, a limitation of their analysis is that bacterial membranes have several components, such as protein channels (porins, efflux, pumps, etc., that were not modeled) that could influence peptide translocation in vivo.

### Efforts to improve lipid bilayer models in our laboratory

In our quest to improve the resemblance of the inner cell membrane of Gram-positive and Gram-negative bacteria we transitioned from our usual binary bacterial model system (POPC:POPG, 8:2), to more complex ones. Based on the work by Epand and Epand 2009, that summarizes phospholipid composition and distribution of these bacteria, we developed two liposome models (PG/PE/PC/CL, 20:50:20:10) and (PG/PC/CL, 40:20:40) mimicking the membranes of Gram-negative and Gram-positive, respectively. Ascaphin- 8 and its amidated isoform were used as the peptides of study. The results obtained reflected the selectivity of the AMPs, with higher affinities toward the Gram-positive model compared to the Gram-negative system. CL induced fluidity as determined by laurdan GP assays. Importantly, membrane surface charge had greater impact than fluidity of peptide activity. To complement the in vitro fluorescence spectroscopy experiments, AA MD simulations were conducted. An important and unforeseen challenge at the time that arose was that the CL reagent was a mixture of various CL species. The mole ratio of these species used in the liposome experiments had to be reasonably represented/mimicked in the in silico approach in order to make the two approaches comparable. Results of the MD simulations revealed that the AMP established stronger electrostatic interactions with CL molecules with greater negative charges. The greater CL concentration also allowed for greater peptide-induced membrane damage. Lastly, area per lipid of the Gram-negative system (less CL) was lower than that of the Gram-positive model, in concordance with liposome fluidity experiments, where lower area per lipid is indicative of a more compact membrane. In terms of thickness, the more compact Gram-negative model was also thicker than the less compact Gram-positive system, consistent with the literature on CL enriched lipid bilayers (Unpublished data). It is important to mention also that, compared to systems without no CL, this lipid had the opposite effect, i.e., increasing rigidity. Thus, depending on lipid composition and environment, CL can increase or decrease membrane fluidity.

## Closing remarks

In the exploration of lipid-protein interactions, the use of membrane models proves essential. To accurately define the activity and specificity of a peptide within a biological membrane, one must not only describe the physicochemical properties of the peptide, which is typically well-documented, but also delve into the physical characteristics of the membrane itself. Most crucially, one needs to understand the intricate relationship between these attributes, as it is this interplay that enables us to define both specificity and selectivity. Refining biological membranes to adequately resemble or mimic that of native cells is not an easy task. This is primarily due to the fact that these models are far from mirroring the native state of cell membranes, as they generally exclude native proteins, glycoproteins/lipids, etc. Moreover, the double membrane of Gram-negative bacteria and the peptidoglycan layer of Gram-positive bacteria have not been successfully modeled together with the main bilayer of these microorganisms. The level of meticulousness in the design depends on the interest of the investigator, i.e., the scientific question or hypothesis that is to be challenged. Most models (generally very simple, three lipid species or less) contribute substantial knowledge on the mechanism(s) of action of membrane-active AMPs. However, it should be a common goal of academic research in this field to improve these models, in pursuance of gaining further insight into how these molecules work. Lipid bilayer models that differentiate between Gram-positive and Gram-negative bacteria are crucial in understanding peptide activity and selectivity. Thus, membrane lipid composition in terms of phospholipid head group and acyl chains, along with asymmetry should be given due consideration as a valuable approach for AMP mechanism elucidation.

## Data Availability

No datasets were generated or analysed during the current study.
